# Multifaceted ORganizational InterventiONs (M-ORION) project for prevention of depression and anxiety among workers: study protocol for a five-arm cluster randomized controlled trial

**DOI:** 10.1186/s12889-024-18112-w

**Published:** 2024-02-24

**Authors:** Kazuhiro Watanabe, Hiroyuki Hikichi, Kotaro Imamura, Asuka Sakuraya, Toru Yoshikawa, Shuhei Izawa, Hisashi Eguchi, Akiomi Inoue, Kengo Yoshida, Yasushi Orihashi, Akizumi Tsutsumi

**Affiliations:** 1 Department of Public Health, Kitasato University School of Medicine, 1-15-1 Kitazato, Minami-Ku, Sagamihara, 252-0374 Japan; 2 Department of Digital Mental Health, Graduate School of Medicine, The University of Tokyo, 7-3-1 Hongo, Bunkyo-Ku, Tokyo, 113-8655 Japan; 3 Research Center for Overwork-Related Disorders (RECORDs), National Institute of Occupational Safety and Health, Japan, 6-21-1 Nagao, Tama-Ku, Kawasaki, 214-8585 Japan; 4 Occupational Stress and Health Management Research Group, National Institute of Occupational Safety and Health, Japan, 6-21-1 Nagao, Tama-Ku, Kawasaki, 214-8585 Japan; 5 Department of Mental Health, Institute of Industrial Ecological Sciences, University of Occupational and Environmental Health, Japan, 1-1 Iseigaoka, Yahatanishi-Ku, Kitakyushu, 807-8555 Japan; 6 Institutional Research Center, University of Occupational and Environmental Health, Japan, 1-1 Iseigaoka, Yahatanishi-Ku, Kitakyushu, 807-8555 Japan; 7 IID Co. Ltd, 4-1-11 Yushima, Bunkyo-Ku, Tokyo, 113-0034 Japan; 8 Clinical Research Center in Hiroshima, Hiroshima University Hospital, 1-2-3 Kasumi, Minami-Ku, Hiroshima, 734-8551 Japan

**Keywords:** Organizational, Psychosocial, Workplace, Mental health, Prevention

## Abstract

**Background:**

Depression and anxiety are the most common mental health issues experienced by workers. Although organizational intervention has been extensively evaluated as a primary prevention of depression and anxiety, the corresponding scientific evidence remains limited because of the lack of cluster randomized controlled trials (cRCT) and failure to detect organizational-level effects. Therefore, the present study aims to assess the preventive effects of four types of interventions on depression and anxiety among workers in an open, five-arm, parallel-group cRCT.

**Methods:**

Overall, 140 worksites and 18,200 nested employees will be recruited from September 2023. The eligible worksites will be randomly assigned to each of the five arms, and programs will be offered for 6–12 months. The five arms are 1) psychoeducation for workers, 2) psychoeducation for supervisors, 3) work environment improvement, 4) physical activity promotion, and 5) active control. The primary outcomes of interest are depression and anxiety. We will also assess psychosocial factors at work, work engagement, health-related quality of life, well-being, economic outcomes, physiological outcomes of health checkups, cortisol levels extracted from fingernails, and indices representing the process and implementation outcomes, including program completion rates. Follow-up surveys will be conducted at 6, 12, and 18 months from baseline, and the primary endpoint is set at the 6-month follow-up. Repeated-measures multi-level mixed modeling will be used to evaluate the effect of each intervention compared with the control.

**Ethics and dissemination:**

The study protocol was approved by the Research Ethics Committee of the Kitasato University Medical Ethics Organization (C22-082). The results and findings of this study will be published in a scientific journal and disseminated to companies that participate in the study.

**Trial registration number:**

UMIN000050949.

**Supplementary Information:**

The online version contains supplementary material available at 10.1186/s12889-024-18112-w.

## Background

Depression and anxiety are the most common mental health conditions among workers. In 2019, 301 and 280 million working-age adults worldwide were living with anxiety and depression, respectively [1]. The 12-month prevalence of major depressive disorder in nationally representative samples of workers was 6.4% and 2.6% in the US [2] and Japan [3], respectively. Many workers also have depression, anxiety, and psychological distress at the sub-clinical level. For example, there are reports showing that 5.2% of workers in the US suffer from depressive symptoms [4], and 9.8% and 8.5% of UK police officers reported depression and anxiety, respectively [5]. The prevalence of psychological distress has been reported to be 4.5% in full-time employees in Australia and 10.8% in employees in Japan [6, 7], and these figures are even higher in some unique populations such as healthcare professionals [8, 9], female sex workers [10], and migrant workers [11]. These mental health issues lead to poor quality of life (QOL) and well-being and increased sickness absence [12], and the global economic cost of mental health issues per year is estimated to be approximately 1 trillion USD, predominantly because of lost productivity [1, 13, 14]. Based on the impact of depression and anxiety on public health and the economy, early intervention and prevention strategies in the context of occupational health are important.

Researchers have extensively reported on the effectiveness of primary (proactive) preventive interventions for mental health conditions [15, 16]. Prior research often applied these interventions at the organizational level to combat major causes of depression and anxiety (e.g., poor psychosocial work environment) among workers [17–19]. There are also systematic reviews on participatory organizational interventions focusing on job design and workload and break changes [20, 21]. Training for supervisors is important in improving their knowledge, attitudes, and behaviors to provide support for their subordinates [22], and academicians have conducted individual-focused interventions to enhance workers’ skills, tolerance, and resilience. The effects of cognitive behavioral techniques, such as mindfulness and cognitive behavioral therapy, or physical activity interventions, such as walking, yoga, resistance training, and aerobics, have been described [16, 23]. Accordingly, a combination of organizational- and individual-focused interventions have been proposed for the effective management of mental health [15, 24].

However, the scientific evidence on these interventions remains limited because of the lack of controlled trials, especially cluster randomized controlled trials (cRCT) [1, 25]. This lack of cRCTs leaves unclear the effects of these interventions, including organizational changes and organizational-level process and implementation outcomes such as feasibility and acceptability [1]. The impact of these interventions on physiological and economic outcomes, such as biomarkers and work performance, also remains underexplored [26]. Further, most previous studies had a short follow-up period; therefore, the long-term impact of the interventions and their sustainability is still unknown [27]. Another research gap relates to the low comparability among interventions. As previous studies have evaluated the preventive effects of each intervention separately in different samples, the most effective intervention has not been identified. These research gaps may lead to ambivalence in employers’ decision-making for choosing appropriate interventions. To address these research gaps, a multi-arm cRCT study with a long follow-up period is needed.

### Objectives

To address these research gaps, the present study aims to assess the effects of four types of organizational interventions on the prevention of depression and anxiety among workers. Non-specific psychological distress will be used as an indicator of depression and anxiety. The interventions include psychoeducation for workers and supervisors, work environment improvement, and promotion of physical activity. The intervention programs contain Internet-delivered or smartphone-delivered content using information communication technologies. As secondary outcomes, the effects on psychosocial factors at work, work engagement, health-related QOL, well-being, economic outcomes, physiological outcomes of a health checkup, and cortisol levels extracted from fingernails will also be identified. Further, the process and implementation outcomes of the intervention programs will be evaluated for their future dissemination in workplaces. Follow-up surveys will be conducted at 6, 12, and 18 months from baseline. The primary hypothesis for this study is that depression and anxiety will significantly improve or be maintained in the four intervention worksites at the primary endpoint (i.e., six months after the baseline) compared with those in active control worksites.

### Trial design

Regarding design, this will be an open, five-arm, parallel-group cRCT study. Of the five arms, four arms will offer intervention programs, and the other will be allocated as an active control group. Randomization will be conducted at the cluster (worksite) level. A worksite is defined as a place where related and systematic work can be conducted in the same place. After completion of the baseline survey, each worksite will be randomly assigned to one of the five arms at a 1:1:1:1:1 ratio. Randomization will be stratified by worksite size, industry, and the percentage of “high-stress” employees in the worksites. Data will be collected at both the worksite and employee levels, and the efficacy of the intervention programs will be evaluated at the employee level, considering the cluster (worksite) level effects. The study protocol has been registered with the University Hospital Medical Information Network (also known as UMIN) Clinical Trials Registry (UMIN-CTR, ID = UMIN000050949). This protocol has been reported in accordance with the SPIRIT guidelines [28].

## Methods

### Study setting

The cRCT will be conducted at worksites in Japan starting September 2023, and the recruitment process will continue until next year. Approximately 2,700 companies that have contracts with IID Co. Ltd. to outsource the National Stress Check Program will be recruited. This program assesses psychosocial stress in employees at least once a year [29]. The potential company participants will cover all regions of Japan, namely Hokkaido, Tohoku, Kanto, Chubu, Kinki, Chugoku, Shikoku, Kyushu, and Okinawa. Based on the Japan Standard Industrial Classification, the potential company participants will cover a wide range of industries, except mining [30].

Invitation flyers will be sent to the companies explaining the background and scientific evidence for organizational interventions to prevent mental illness in the workplace as well as the objectives of the present study. Stressful worksites will be prioritized for recruitment to clearly detect the effects of the interventions using the percentage of “high-stress” employees from the previous results of the National Stress Check Program. The definition of “high-stress” is based on a combination of high scores in stress response and job stressors, and low scores in social support. The predictive validity of “high-stress” employees for long-term sickness absence at a one-year follow-up has been reported previously [31].

**Fig. 1:**
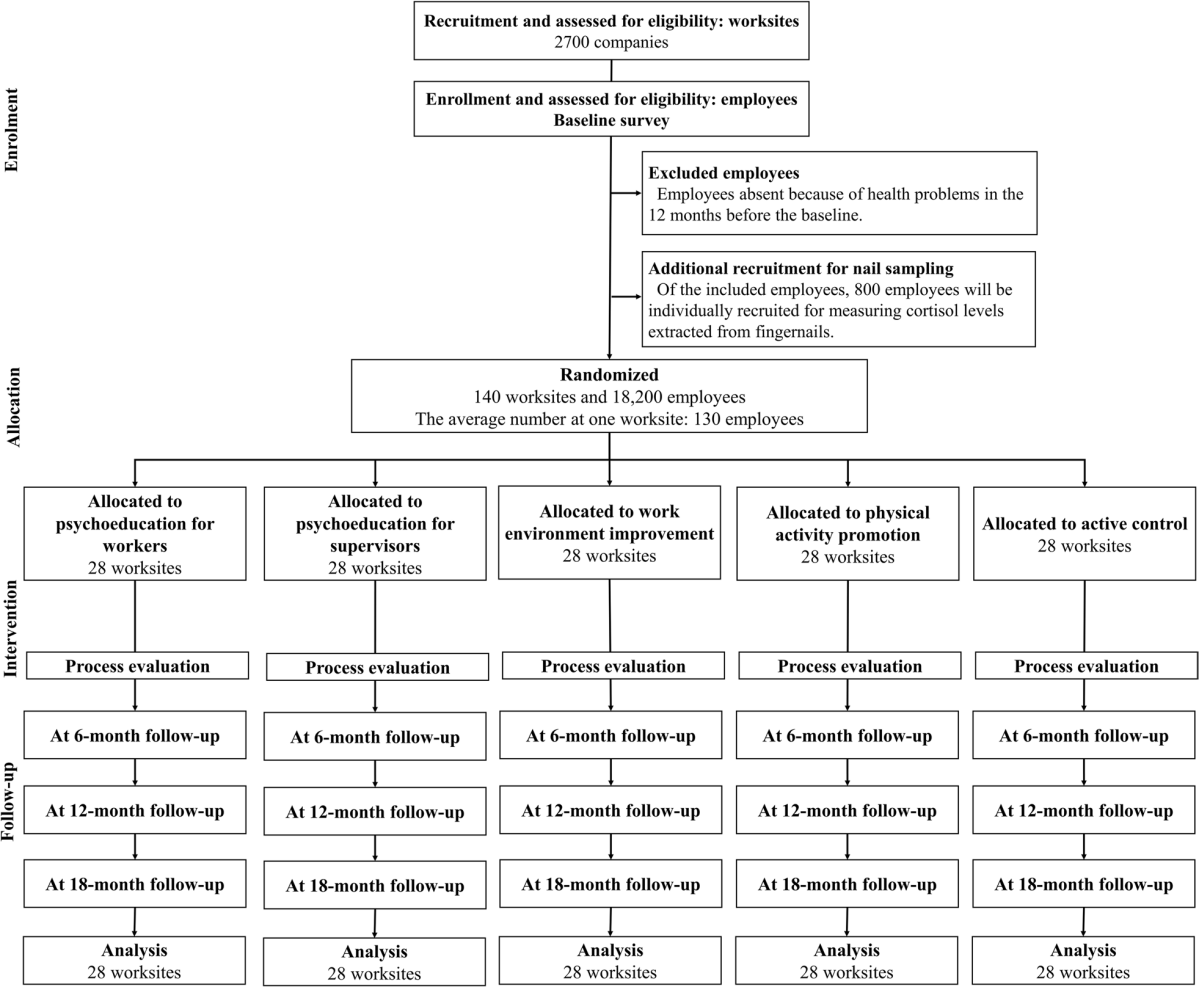
Participant flow chart

Figure 1 presents the participant flowchart of this study. When the company agrees to participate, a list of worksites will be provided to the research team with information on location, worksite size, industry, and contact details. All employees nested within the worksites meeting the eligibility criteria will be recruited. Informed consent will be obtained from all employees at the baseline survey via paper-signed or online forms. After completion of the baseline survey, the worksites will be randomly allocated to one of the five arms. The intervention programs will last for 6–12 months. The follow-up surveys will be conducted 6, 12, and 18 months after the baseline survey. Of the included employees, 800 will be individually recruited for quantification of cortisol levels extracted from fingernails. These employees will be asked to clip extended fingernails into a zip-lock plastic bag and answer a questionnaire about lifestyle habits associated with fingernails in each of the surveys.

### Participants

This cRCT will recruit multi-level participants, including worksites and employees. The inclusion criteria for worksites are 1) conducting the National Stress Check Program; 2) analyzing the results of the National Stress Check Program in sufficiently large groups for work environment improvement [29]; 3) employing 5–299 workers subject to the National Stress Check Program; 4) having at least one supervisor working with at least one subordinate. There are no exclusion criteria for worksites. The inclusion criteria for employees are 1) being aged ≥ 18 years; 2) being able to answer the questionnaires written in Japanese; 3) having access to the Internet and the intervention programs offered online; 4) having a private smartphone available; 5) working at a workplace (or workplaces) where at least one supervisor and one subordinate work together. The exclusion criterion for employees is absence from the workplace because of health problems in the 12 months before baseline. The inclusion/exclusion criteria for employees at the participating worksites, including teleworkers, do not involve other work parameters, such as employment, shift, and occupational types. Additional exclusion criteria are set for 800 employees who will be providing samples for quantification of cortisol levels extracted from fingernails: 1) participants with fingernails shortened using only a nail file (not cutting fingernails) and 2) participants using false nails.

### Interventions

This cRCT will offer four intervention programs and an active control program at the worksite level. The interventions include psychoeducation for workers and supervisors, work environment improvement, and physical activity promotion (Supplementary File 1).

#### Psychoeducation for workers

Workers in the worksites allocated to this intervention group will be asked to use a web-based app that provides psychoeducation on job crafting for six months. The participants will access the contents by smartphones, tablets, or computers. The contents of the app were based on the theory of job crafting, “the physical and cognitive change individuals make in the task or relational boundaries of their work,” as defined by Wrzesniewski and Dutton [32]. It was developed mainly based on a previous face-to-face job crafting program [33], and includes three types of job crafting (i.e., task, relation, and cognition).

The education program on job crafting contains four sessions, each lasting 10–15 min. First, the app provides lectures about the concept and importance of job crafting. Second, it introduces various examples of job crafting, such as how workers change their tasks or relationships in their working lives to be more desirable. Third, it requires participants to create their own job crafting plans and try them out. After that, they look back at their plan and make a new improved version of the plan. Each session can be taken at any time and at the participants’ convenience. During the intervention period, participants will be recommended to try various job crafting plans by using the app. If they have any questions or comments about the app, they will be able to contact researchers by e-mail. To encourage participants to use the app, a reminder e-mail by the researcher will be sent once a week for the first month, and once a month from the second to the sixth month.

#### Psychoeducation for supervisors

Supervisors at the worksites allocated to this arm will be asked to take an online-based training program named “Maximize Team Capabilities! Workplace Psychological Safety Navigation” for six months. The program is mainly based on the rationale of team psychological safety by Edmondson [34]. Psychological safety is defined as a shared belief that the team is safe for interpersonal risk-taking (i.e., doing learning behavior that may place workers at risk, including seeking feedback, sharing information, asking for help, talking about errors, and experimenting).

This program provides an explanation of the rationale and importance of team psychological safety at work, how to promote team psychological safety, and specific actions for supervisors to build team psychological safety. The program also provides information on how to support subordinates who may be experiencing mental problems or concerns (i.e., how to notice changes in their health, approach them, and encourage consultation with mental health professionals). This program comprises four video lessons and e-newsletters. The videos will be released on YouTube, one per week during the first month. The videos are approximately 15 min each and can be viewed as many times as participants like. The e-newsletter will be delivered three times a week during the first month, and then once a week from the second month to the sixth month. The e-newsletters offer lesson summaries, encourage viewing for those who have not watched, and provide specific examples of actions to promote team psychological safety in the workplace.

#### Work environment improvement

Workers in the worksites in this intervention group will participate in the Participatory Work Environment Improvement Program (PWIP) [35] for 7–12 months, which focuses on improving the psychosocial factors of the workplace. It is based on participatory organizational interventions and participatory approaches that are undertaken to respond to local needs at each workplace [36]. This program will be conducted by internal facilitators who will be selected in each workplace and will assess, facilitate, and provide suggestions for the PWIP. The external coordinators (i.e., the research team) will contact, train, and support the internal facilitators to conduct the PWIP program.

First, a single, 120-min training session for the internal facilitators will be held immediately after allocation. Next, the internal facilitators will assess the readiness of the PWIP by using the Basic Organizational Development for Your workplace (also known as BODY) checklist [37]. This checklist consists of five items and classifies readiness of the workplace into four levels (from 0–4). The PWIP will be tailored based on the proportion of workers with affirmative answers to the Basic Organizational Development for Your workplace items, as follows: for a workplace classified as level 2 or 3, the standard PWIP program will be conducted; for a workplace classified as level 0 or 1, the external coordinators increase the frequency of contact and support for the internal facilitators. The planning and application of the PWIP will be supported by a web-based app. Workers will access the app to identify the favorable conditions and conditions that need improvement in the workplace, participate in group work, and contribute to the improvement of the workplace [38]. The PWIP consists of three sessions, as described herein: 1) a kick-off work (first time), encompassing a brief workplace-level workshop, which in turn uses a) presentations of local good examples, b) action checklists listing typical low-cost actions [39], c) group work guidance materials on the website for job stress prevention; 2) follow-up/planning and actions (1–3 months later), including workplace-level feasible multifaceted improvement plans, which in turn will be devised by encouraging consensus building and facilitating actions at each work unit or workplace; 3) report and shared achievements (6–12 months later), emphasizing locally-achieved good practices that improved working conditions and worker safety and health.

#### Physical activity promotion

Workers in the worksites in this intervention group will be asked to install a smartphone app on their smartphone and use it for six months. The app is named ASHARE, and is a native app for iOS and Android smartphones, supporting version 12.0 or later in iOS and version 5.0 or later in Android [40]. The iOS and Android versions of the app are distributed via App Stores and Google Play, respectively.

The app was developed to promote physical activity and prevent depression and anxiety in a healthy working population, and is based on basic behavioral change techniques (i.e., self-monitoring, feedback, and sharing of users’ physical activity data). Moreover, the needs of workers identified through qualitative interviews in a past study were reflected in the functions and interface of the app [41]. When users install the app, they are initially asked to register their username and email address and input their age, gender, occupation, employment status, shift type, working hours per week, holiday patterns, and preferred activities. The app works with Apple Healthcare (iOS) and Google Fit (Android) and obtains data on the duration of moderate-to-vigorous physical activity every 15 min [42]. These data are stored on a cloud server. The duration of the overall physical activity on the previous day is depicted as a graph on the top screen of the app.

In addition, the deep-learning model embedded in the app accesses the data for the previous day on cloud servers when the user starts the app and predicts a score of psychological distress for the day, which in turn is measured using the six-item Kessler Psychological Distress Scale (K6) [43]. Every day, participants are provided with feedback on predicted scores and levels of distress, which are presented as weather conditions, accompanied by rule-based comments from a monkey anime character (mental health forecast). A previous study on the predictive performance of the deep-learning model underlying the ASHARE app reported that the correlation coefficient between the predicted and measured values for psychological distress was 0.679 (R2 = 0.463), and that the overall classification accuracy for levels of psychological distress was 76.3% [44]. The predicted scores and levels of psychological distress are recorded and monitored on a monthly basis.

The ideal usage of the app, as expected by the researchers, is for the participants to take about 5 min every day to open the app, check their physical activity patterns and the results of their mental health forecast, and to then increase the amount of any type of moderate-to-vigorous physical activity. As a function of data sharing, users with a high percentage of sunny days are ranked as “best performers.” Rankings can be modified freely based on categories such as age, gender, occupation, and preferred activities. If users are reluctant to share data with other users, they can decline to participate in the ranking process. The app has a notification function that notifies users on the smartphone of new information and the result of the mental health forecast (provided at six o’clock every morning). The reminders for app use will be sent once a week using this function.

#### Active control

Workers in the worksites in this group will be asked to download and read a booklet with basic information about stress, and to complete a simple individual work for six months. The booklet is a 14-page PDF file comprising explanations about differences between stressors and stress reactions (or strain); stress-performance curve (i.e., Yerkes–Dodson law) [45]; stressful life events [46]; and National Institute for Occupational Safety and Health (also known as NIOSH) model of job stress [47]. It also includes a worksheet to write down the acute reactions (i.e., psychological, physiological, and behavioral) that are likely to occur when the participants themselves are placed in stressful situations. Since this is a control group, as a rule, no assistance other than the distribution of the booklet will be provided.

#### Strategies to improve adherence to intervention protocols

To improve adherence (completion rates) to the programs, reminders will be sent to participating worksites and employees via email and/or applications during the program duration. The reminders will include greetings, a recommendation to work on the contents of assigned programs, and the sharing of the statistics of the completion rates at other worksites within the arms (e.g., mean rates and champion worksite). Completion rates will be monitored using the digital records of the intervention and active control programs. At each survey point, reminders to complete the questionnaire will be sent weekly. These process evaluations of the cRCT will be summarized immediately after each survey (at the 6-, 12-, and 18-month follow-ups) and sent to the participating worksites and employees with a letter thanking them for their cooperation.

### Outcomes

Table 1 presents a schedule of enrolment, interventions, and assessments of this cRCT. All outcomes will be measured at the employee level. As the primary outcomes, depression and anxiety will be measured at baseline and at the 6-, 12-, and 18-month follow-ups. As secondary outcomes, psychosocial factors at work, work engagement, health-related QOL, well-being, economic outcomes, physiological outcomes from a health checkup, and levels of cortisol extracted from fingernails will be measured. The process and implementation outcomes will be assessed as well.

**Table 1:**
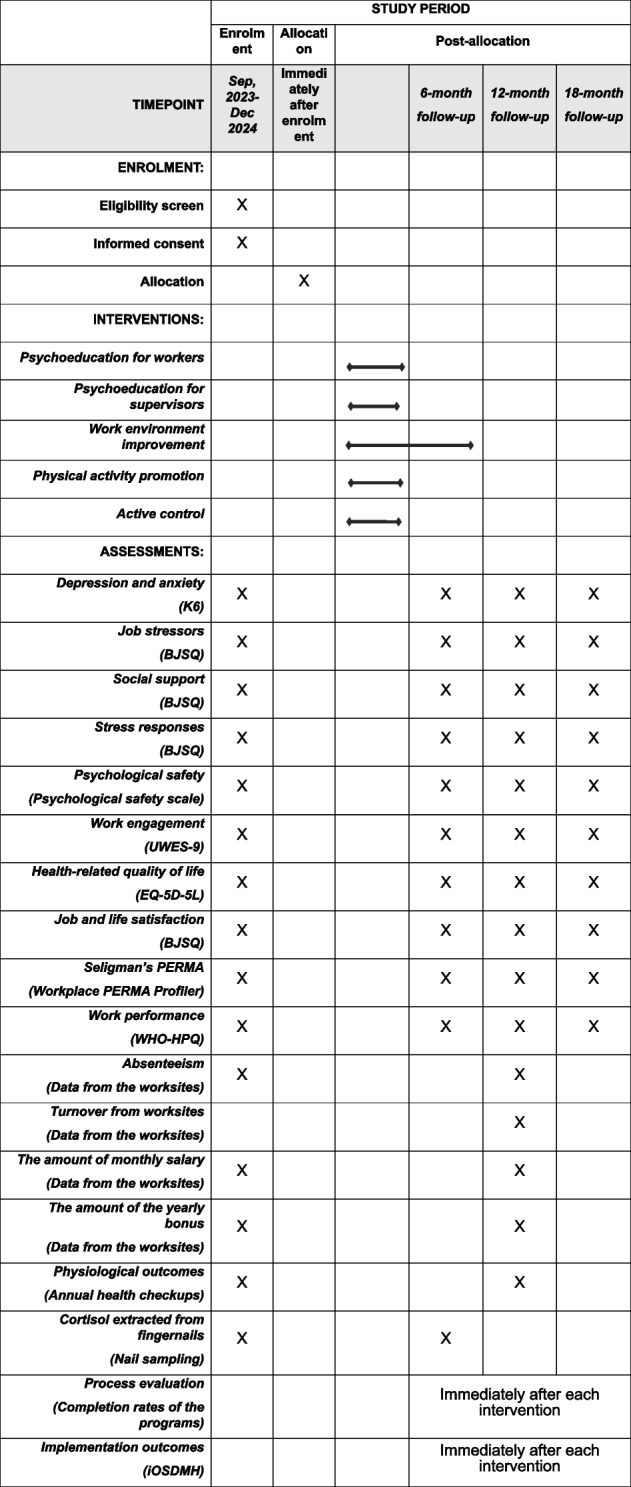
The schedule of enrolment, interventions, and assessments

*BJSQ* Brief Job Stress Questionnaire, *EQ-5D-5L* the Japanese five-level version of the scale by the EuroQoL group, *UWES* Utrecht Work Engagement Scale, *iOSDMH* Implementation Outcome Scales for Digital Mental Health, *K6* Kessler Psychological Distress Scale, *WHOHPQ* World Health Organization Health and Work Performance Questionnaire

#### Depression and anxiety

Depression and anxiety (non-specific psychological distress) will be measured using the Japanese version of the K6 scale [43]. It comprises six items that assess the frequency of experience of non-specific psychological distress, which are rated on a five-point Likert scale (0 = none of the times and 4 = all the times). The reliability and validity of the Japanese version of the K6 have been confirmed previously [43]. In this study, the total score for the K6 scale will be considered an indicator of depression and anxiety.

#### Psychosocial factors at work

Job stressors, stress responses, and psychological safety will be measured as indicators of psychosocial factors at work. Job stressors include job demands, job control, and social support from supervisors and coworkers, which will be measured using subscales of the Brief Job Stress Questionnaire (BJSQ) [48]. The BJSQ was developed in 2000 in Japan and has been widely used in occupational health studies and practice [49]. The subscales for job stressors comprise three items each and are job demands, job control, supervisor support, and coworker support. All items are rated on a four-point Likert scale (for job demands and job control: 1 = not at all, 4 = very much so; for supervisor and coworker support: 1 = not at all, 4 = extremely). The total subscale scores will be calculated.

Stress responses will also be measured using the BJSQ, and the subscales are vigor (three items), anger-irritability (three items), fatigue (three items), anxiety (three items), depression (six items), and physical complaints (11 items). All items are rated on a four-point Likert scale (1 = almost never, 4 = almost always). The total subscale scores will be calculated.

Psychological safety will be measured using the Japanese version of the Psychological Safety Scale (also known as PSS) [50]. It assesses workers’ perceptions of the consequences of interpersonal risks in the workplace and consists of three subscales: team leader (nine items, e.g., “If I had a question or was unsure of something in relation to my role at work, I could ask my team leader”), peers (seven items, e.g., “I can communicate my opinions about work issues with my peers”), and team as a whole (three items, e.g., “It is easy to ask other members of this team for help”) [51]. All items are rated on a seven-point Likert scale (1 = strongly disagree, 7 = strongly agree). The total subscale scores will be calculated.

#### Work engagement

The nine-item Japanese version of the Utrecht Work Engagement Scale (also known as UWES) will be used to assess work engagement [52]. It consists of three subscales, namely vigor (three items, e.g., “At my job, I feel strong and vigorous”), dedication (three items, e.g., “I am enthusiastic about my job”), and absorption (three items, e.g., “I am immersed in my work”). All items are rated on a seven-point Likert scale (0 = never, 6 = always). The reliability and unidimensional validity of the Japanese version of this scale have been confirmed previously [52]. The average scores from each of the nine items will be used for analyses.

#### Health-related QOL

Health-related QOL will be measured using the Japanese five-level version of the scale by the EuroQoL group (also known as EQ-5D-5L) [53]. It consists of five dimensions of QOL (mobility, self-care, usual activities, pain or discomfort, and anxiety or depression). Each dimension has five levels for perceived problems, and a higher score indicates a higher QOL. We will use the value set of the Japanese version of this scale that has been developed in previous research [54].

#### Subjective well-being

Job and life satisfaction will be measured as indicators of subjective well-being using questions from the BJSQ [48, 49], with one item for each: “I am satisfied with my job” and “I am satisfied with my family life,” respectively. The two items are rated on a four-point Likert scale (1 = dissatisfied, 4 = satisfied), with higher scores indicating higher satisfaction.

The Japanese version of the Workplace PERMA Profiler will also be used as a multidimensional concept of well-being [55]. This scale is based on Seligman’s PERMA model, and consists of five domains: positive emotion (P), engagement (E), relationships (R), meaning (M), and accomplishment (A) [56]. An overall score of well-being at work is calculated as the average of 15 items (three items each for five dimensions) and happiness (one item). All items are rated on an 11-point Likert-type scale (ranging from 0–10; e.g., for the first item of positive emotion, 0 = never and 10 = always). The reliability and validity of the Japanese Workplace PERMA Profiler have been confirmed previously [55].

#### Economic outcomes

Economic outcomes include work performance, absenteeism, worksite turnover, and monthly salary and yearly bonuses. Work performance will be assessed using an item for the absolute score of work performance from the Japanese version of the World Health Organization Health and Work Performance Questionnaire short version (also known as WHOHPQ) [57]. The item rates an individual’s overall job performance in the past month on a scale of 0‒10 (0 = worst job performance, 10 = best job performance). The ratings are multiplied by 10 to calculate work performance according to the scoring guidelines.

Regarding absenteeism, data on absent days in the year before the surveys, monthly salary, and yearly bonus will be obtained from the worksite database at the time of the baseline survey and the 12-month follow-up survey. Regarding worksite turnover, data on the employees leaving the worksite will be obtained at the 12-month follow-up survey.

#### Physiological outcomes from a health checkup

Data on physiological outcomes, based on the annual health checkups conducted by the participating worksites, will also be collected at baseline and at the 12-month follow-up surveys. These outcomes include height, weight, waist circumference, blood pressure, blood lipids, and blood glucose levels.

#### Cortisol extracted from fingernails

In total, 800 employees will be asked to clip extended fingernails into a zip-lock plastic bag for one month, and cortisol levels will be measured from the nail samples. This procedure will be conducted at baseline and at the 6- and 18-month follow-ups. During sample collection, health conditions and lifestyle topics associated with cortisol levels and fingernails will be assessed using a self-reported questionnaire. The questionnaire will include items on marital status, educational background, income, medical history, current medications, pregnancy/childbirth in the previous year, smoking and drinking habits, frequency of cutting nails, frequency of using manicures, how to shorten nails (cut only or cut and file), habit of biting nails, frequency of soap use, frequency of hand disinfection, and frequency of kitchen detergent use.

#### Process and implementation outcomes

For process evaluation, the completion rates of the intervention and active control programs will be collected from the digital records of the programs. Implementation outcomes will be assessed using the Implementation Outcome Scales for Digital Mental Health (also known as iOSDMH), which was shown to have acceptable reliability and validity [58]. This assessment tool was developed to measure the implementation outcomes of mental health interventions delivered by digital and telecommunication technologies, and comprises the following five subscales: acceptability (three items), appropriateness (four items), feasibility (six items), overall satisfaction (single item), and harm (five items). These evaluations will be conducted immediately after each intervention.

### Sample size calculation

The required sample size has been calculated according to the guidelines of the Consolidated Standards of Reporting Trials (also known as CONSORT) for cRCTs [59], considering the intraclass correlations of the outcomes nested by the worksites. The sample sizes in cRCTs should be multiplied by the design effect (1 + [m-1]ρ), where m is the average cluster size and ρ is the intraclass correlations [60]. In the dataset from a cohort of healthy Japanese workers [61, 62], the intraclass correlation for depression and anxiety measured by the K6 scale was 0.013. Therefore, a very small intraclass correlation (0.05, 5% of the outcome would be shared at the worksite level) was estimated for the primary outcome in this study. The average cluster size (the number of employees within one worksite) was set to 130 based on the information about potentially recruiting 2,700 companies from IID Co. Ltd.

The effect size of the four intervention programs on the primary outcome, that is, depression and anxiety, was estimated to be 0.20 in the standardized mean difference (d). A previous RCT that offered an Internet-based cognitive behavioral approach to prevent depression among nonclinical workers reported the effect size (d) of the intervention as 0.20 at the 6-month follow-up [63]. The effect sizes of work environment improvement and physical activity promotion on mental health among nonclinical workers were reported to be larger [35, 64]. An alpha error probability (α) has been set to 0.05/4 = 0.0125 (one-tail) because we plan four tests between the intervention programs and control programs. The statistical power (1-β) has been set to 0.80.

Based on these parameters, the required sample size has been calculated as 3,554 for each arm using G* Power version 3.1.9.2 [65]. Considering that the average number of employees within one worksite is assumed to be 130, 27.3 worksites will be needed in each arm. Therefore, we have set the required number of worksites and employees to 28 and 3,640 in each arm and to 140 and 18,200 in total.

The required number of worksites (140) is 5.2% of the recruited companies (2,700). Previous RCTs on nonclinical workers at multiple worksites in Japan reported acceptance rates of approximately 4% [63, 66]. In this cRCT, the acceptance rate is likely to be higher and to reach the target sample size by prioritizing the recruitment of stressful worksites, which may in turn require countermeasures for stress management.

### Randomization

Each included worksite will be randomized to one of the five arms. The randomization will be stratified into 3 × 2 × 2 = 12 strata, based on worksite size (three strata, 5–49, 50–99, and 100–299 employees), industries (two strata, manufacturing/construction/transportation, and other industries), and the percentage of “high-stress” employees in the worksites (two strata, < 15% and ≥ 15%). Companies that engage in manufacturing/construction/transportation are considered qualitatively different from other companies because they exert a physical demand on the workers and focus on reducing work-related injuries. The cutoff point for the percentage of “high-stress” employees in the worksites was decided according to previous results of the National Stress Check Program in the potentially participating companies. Permuted block randomization (block size = 5) will be adopted for equal randomization. A stratified permuted block random table will be created by an independent biostatistician at the Department of Clinical Research, Kitasato University Hospital. This table will be managed by another research assistant in the Department of Public Health, Kitasato University School of Medicine, who will be blinded to researchers. The results of randomization will be open to the participating worksites and employees because the contents of all intervention programs will be notified before enrollment. The assessment of the outcomes will also be open because of the self-assessment of depression and anxiety. Data analysis will be blinded because it will be conducted by the second author (HH), who will not participate in the recruitment, enrollment, allocation, and delivery of the intervention programs.

### Statistical analysis

For the main analysis, repeated-measures multi-level mixed modeling will be used to test the effects of each intervention compared with the control at the 6-, 12-, and 18-month follow-ups. The interaction effect of the group (1 = intervention, 0 = control) by time will be considered an indicator of the intervention effect. The multi-level is based on employees (level 1) and worksites (level 2). The variables used in the stratified randomization (i.e., worksite size, industries, and the percentage of “high-stress” employees in the worksites) will be used as covariates. Intention-to-treat analysis will be adopted by including all employees who completed the baseline survey in the analysis. Multiple imputations will be conducted for missing values owing to dropouts. Effect sizes will be calculated as the standardized mean differences (d) of the scores on the K6 scale, based on the estimated mean differences.

The primary endpoint of the primary outcomes is set at the 6-month follow-up, and the corresponding statistical significance will be the primary indicator of effectiveness. If recruitment does not achieve the target sample size (140 worksites and 18,200 employees in total), we will assess the intervention effectiveness based on a minimally important difference. As the estimated effect size of the interventions is 0.20, the distribution-based approach [67] is useful for estimating the minimally important difference of depression and anxiety measured by the K6 scale. In a previous RCT among nonclinical workers in Japan [63], the standard deviation of the change in the K6 score at the 6-month follow-up was 4.16. From the distribution, 20% of the standard deviation is estimated to be 0.832. Therefore, a 0.85 score change in the K6 score at the 6-month follow-up is considered to be the minimally important difference.

The secondary and continuous outcomes will be analyzed using the same method as the primary outcomes, that is, multi-level mixed modeling. At the 12-month follow-up, the prevalence rate of worksite turnover in each intervention group will be compared with that in the control group using the logistic regression model. The means of the completion rates of the programs and the scores of implementation outcomes will be compared among the five arms using analysis of covariance. Per protocol set analyses will be conducted assuming that some participants may not complete all protocols of the intervention programs. All statistical analyses will be conducted using STATA version 17 (StataCorp LLC, Texas, USA).

### Data monitoring

A data monitoring committee will be established comprising the corresponding author (KW), the manager of enrollment (KY), and intervention program providers (AS, KI, TY, KW, and AI). The data monitoring committee meeting will be held every six months, in line with the surveys. The purpose of the meetings will be to review the recruitment process, program completion rates, attrition rates, and any harm caused by the interventions.

## Ethics and dissemination

### Ethical considerations

The study protocol was approved by the Research Ethics Committee of the Kitasato University Medical Ethics Organization, Japan (C22-082). Informed consent will be obtained from representatives of the worksites when a company agrees to participate in the study. In the baseline survey, consent from employees will also be obtained via paper-signed or online forms. If eligible employees in the participating worksites refuse to join the study, we will not include them in the study but will allow them to be offered interventions. The obtained data will be stored on a standalone computer at the Department of Public Health, Kitasato University School of Medicine, and managed by an independent research assistant. The data will not be anonymized and shared with the intervention program providers within the research team on request when the implementation data are analyzed or reminders and feedback with letters of gratitude are sent. The research ethics committee approved the data management plan.

### Dissemination of research findings

The results and findings will be submitted and published in a peer-reviewed scientific journal according to the Consolidated Standards of Reporting Trials guidelines for cRCTs [59]. The results will also be provided to the participating worksites and employees. If the effectiveness and implementation outcomes are significant and promising, intervention programs will be packaged by private vendors in Japan and disseminated to companies that do not participate in the study.

### Strengths and limitations

This study will be the first cRCT with a large sample size, multiple arms, and long follow-up period to investigate the effect of primary preventive interventions on depression and anxiety in occupational health. The results and findings from the 140 worksites and 18,200 employees will be generalizable to other worksites in Japan. Moreover, the multiple-arm and outcome-wide study design will enable us to compare the effectiveness of the four interventions on broad outcomes simultaneously, with the same control group. Therefore, the results and findings will establish a model to present the scientific evidence and support the decision-making of employers for future primary preventive approaches.

This study will have several limitations. In the long follow-up period, some employees and some worksites will likely be removed from the study. Although we will adopt intention-to-treat analysis and plan to impute the missing values at the analysis phases, too much attrition will cause selection bias. Moreover, as the primary outcomes, depression and anxiety, will be measured using a self-reported scale, the results for the effectiveness of the programs will contain certain measurement errors and information bias. Furthermore, the randomization of the five arms will be conducted under various strata, meaning that even if a large pool of worksites is included, successful randomization depending on recruitment cannot be guaranteed.

### Supplementary Information

**Supplementary Material 1.**

## Data Availability

No datasets are available because this is the study protocol of a trial to be conducted in the future.

## Abbreviations

BJSQ: Brief Job Stress Questionnaire
cRCT: Cluster randomized controlled trial
K6: Kessler Psychological Distress Scale
PWIP: Participatory work environment improvement program
QOL: Quality of life
