# Supplementary material for: Multifaceted ORganizational InterventiONs (M-ORION) project for prevention of depression and anxiety among workers: study protocol for a five-arm cluster randomized controlled trial
Source: BMC Public Health. 2024 Feb 24;24:601. doi: 10.1186/s12889-024-18112-w (PMC10894478; doi:10.1186/s12889-024-18112-w)
Supplement: Supplementary file 1 — Supplementary Material 1. [file 12889_2024_18112_MOESM1_ESM.docx]

**Supplementary File 1.** Intervention programs and an active control program that this trial will offer

The screenshots of each program are uploaded to our website.

<https://www.med.kitasato-u.ac.jp/lab/publichealth/M-ORION_supplementary1.pdf>

Note. Because these programs were developed for Japanese workers (not English natives), the contents are written in Japanese.
